# Necroptosis of hippocampal neurons in paclitaxel chemotherapy-induced cognitive impairment mediates microglial activation via TLR4/MyD88 signaling pathway

**DOI:** 10.1515/med-2025-1182

**Published:** 2025-05-02

**Authors:** Lan-Lan Liu, Xin Liu, Shuang Zhao, Zhao Li, Jia-Xin Liu, Dong-Yang Ma, Xiu-Li Wang

**Affiliations:** Department of Anesthesiology, The Third Hospital of Hebei Medical University, Shijiazhuang, Hebei, 050051, P.R. China; Department of Anesthesiology, The Third Hospital of Hebei Medical University, No. 139 Ziqiang Road, Shijiazhuang, Hebei, 050051, P.R. China

**Keywords:** paclitaxel, necroptosis, DAMPs, microglia polarization, TLR4/MyD88 signaling pathway

## Abstract

**Background:**

Paclitaxel (PTX) chemotherapy frequently induces cognitive impairment, which is closely associated with two key pathological processes: necroptosis of hippocampal neurons and microglial polarization. Necroptotic neurons release damage-associated molecular patterns, triggering inflammatory responses. As the primary immune cells in the central nervous system, microglia can exhibit either pro-inflammatory or anti-inflammatory activity depending on their polarization state. However, the relationship between PTX-induced neuronal necroptosis and microglial activation remains unclear.

**Methods:**

In this study, both *in vivo* and *in vitro* experiments were conducted. *In vivo*, an adult male C57BL/6N mouse model of PTX-induced cognitive impairment was established and divided into three groups: Veh (vehicle control), PTX (paclitaxel only), and P + N (paclitaxel with Nec-1 treatment). Necrostatin-1 (Nec-1), a specific inhibitor of RIPK1, was used to inhibit necroptosis. *In vitro*, HT22 cells were used to prepare necroptosis-conditioned medium, and BV-2 cells were treated with this medium. TAK-242, a TLR4 inhibitor, was used to explore the role of the TLR4/MyD88 signaling pathway. Immunofluorescence staining, western blot, and ELISA were employed to detect relevant markers and cytokines.

**Results:**

The results demonstrated that PTX-induced necroptosis of hippocampal neurons activated microglia. Nec-1 effectively suppressed neuronal necroptosis and reduced M1 polarization of microglia. The TLR4/MyD88 signaling pathway was involved in microglial polarization induced by the necroptotic-conditioned medium of PTX-treated HT22 cells. TAK-242 significantly blocked the regulatory effect of PTX-induced neuronal necroptosis on BV-2 microglial polarization.

**Conclusion:**

This study reveals that hippocampal neuron necroptosis activates microglia through the TLR4/MyD88 signaling pathway in PTX-induced cognitive impairment, promoting M1 polarization and neuroinflammation. Inhibiting necroptosis promotes M2 polarization and neuroprotection. These findings uncover a novel mechanism of PTX-induced cognitive impairment and suggest potential therapeutic targets.

## 1 Introduction

Paclitaxel (PTX) is a widely used first-line chemotherapy drug. While it effectively kills cancer cells, PTX often impairs patients’ cognitive functions, including learning and memory, a condition commonly referred to as “chemo-brain” [1]. Among breast cancer patients receiving PTX chemotherapy, 15–75% report mild to moderate cognitive impairment, with 35% experiencing persistent symptoms months or even years after treatment [1]. Previous studies have explored potential strategies to prevent or reverse PTX-induced cognitive decline [2,3]. However, these strategies still face challenges in clinical translation, and a more in-depth understanding of the underlying mechanisms and the development of more effective early intervention methods remains urgent needs.

Necroptosis, a form of programmed cell death mediated by receptor-interacting protein kinases 1 and 3 (RIPK1/3) and mixed lineage kinase domain-like protein, has been implicated in PTX-induced cognitive impairment [4,5]. Our prior research demonstrated that necroptosis of hippocampal neurons plays a crucial role in this condition. Using Necrostatin-1 (Nec-1), a specific RIPK1 inhibitor, we successfully inhibited necroptosis and improved cognitive function in a mouse model of PTX-induced cognitive impairment.

While cell death is traditionally viewed as a consequence of inflammation, recent evidence suggests that certain forms of cell death, such as necroptosis, can actively exacerbate inflammatory responses [6]. Unlike apoptosis, which releases minimal damage-associated molecular patterns (DAMPs), necroptosis releases significant amounts of DAMPs, recognized by pattern recognition receptors (PRRs) that amplify immune responses by upregulating chemokines and cytokines [7,8]. In neurodegenerative diseases, DAMPs activate microglia and initiate neuroinflammatory responses. As the brain’s resident immune cells, microglia continuously monitor the central nervous system for changes in the microenvironment [9,10].

Microglia exhibit functional plasticity, adopting distinct phenotypes under various pathophysiological conditions. The M1 pro-inflammatory phenotype releases neurotoxic factors, exacerbating neuronal damage and contributing to cognitive and memory deficits [11–14]. In contrast, the M2 anti-inflammatory phenotype promotes neuronal survival by releasing neurotrophic factors and clearing cellular debris, offering neuroprotection [15,16]. Evidence increasingly highlights the central role of microglial-mediated neuroinflammation in the progression of neurodegenerative diseases [17]. Our previous work showed that M1 microglial polarization contributes significantly to PTX-induced neurological damage, serving as a primary mechanism of both cognitive impairment [18] and neuropathic pain [19]. Therefore, investigating the role of DAMPs released by necroptotic hippocampal neurons in microglial polarization may uncover novel targets for preventing and treating PTX-induced cognitive impairment.

Toll-like receptors (TLRs) are key PRRs expressed on microglia, mediating innate immune responses and modulating inflammation following central nervous system injury [20]. Myeloid differentiation factor 88 (MyD88) is a critical adapter protein in TLR signaling pathways, including TLR4, IL-1R, and IL-18R, and activates downstream effectors such as NF-κB and MAPK to promote inflammation [21,22]. Among TLRs, TLR4 has been closely linked to central nervous system inflammation [23–27]. TLR4 inhibition has demonstrated neuroprotective effects in models of doxorubicin-induced cognitive impairment [28] and cerebral ischemia [29]. Additionally, recent studies indicate a connection between TLR4 signaling and necroptosis in macrophages and tumor cells [30–33]. However, whether PTX-induced necroptosis of hippocampal neurons regulates microglial polarization through the TLR4/MyD88 pathway remains unclear.

This study explores, for the first time, the role of DAMPs derived from hippocampal neurons in activating microglia via the TLR4/MyD88 pathway. These findings are expected to provide new insights and therapeutic targets for PTX-induced cognitive impairment.

## 2 Materials and methods

### 2.1 Establishment of PTX-induced cognitive impairment model in mice and treatment protocol

Adult male C57BL/6N mice (6–8 weeks old, 20–24 g, SPF grade) were purchased from Beijing Sibeifu Laboratory Animal Technology Co., Ltd (License No.: SCXK (Beijing) 2016-0006). The study was approved by the Experimental Animal Welfare Ethics Committee of Hebei Medical University (Ethics No.: IACUC-Hebmu-2020009). After 1 week of acclimation, the mice were randomly assigned into three groups (*n* = 8 per group) using a random number table. A previously established PTX-induced cognitive impairment model was employed. The treatment methods and dosages for each group were as follows.

### 2.2 Drug dosage selection

The doses of PTX (10 mg/kg) and Nec-1 (6.5 mg/kg) were chosen based on previous studies demonstrating their efficacy in murine models of chemotherapy-induced neurotoxicity [34,35]. These doses were shown to induce significant neuronal necroptosis and cognitive impairment (for PTX) or effectively inhibit RIPK1 activity (for Nec-1) without causing overt systemic toxicity.

**Figure 1:**
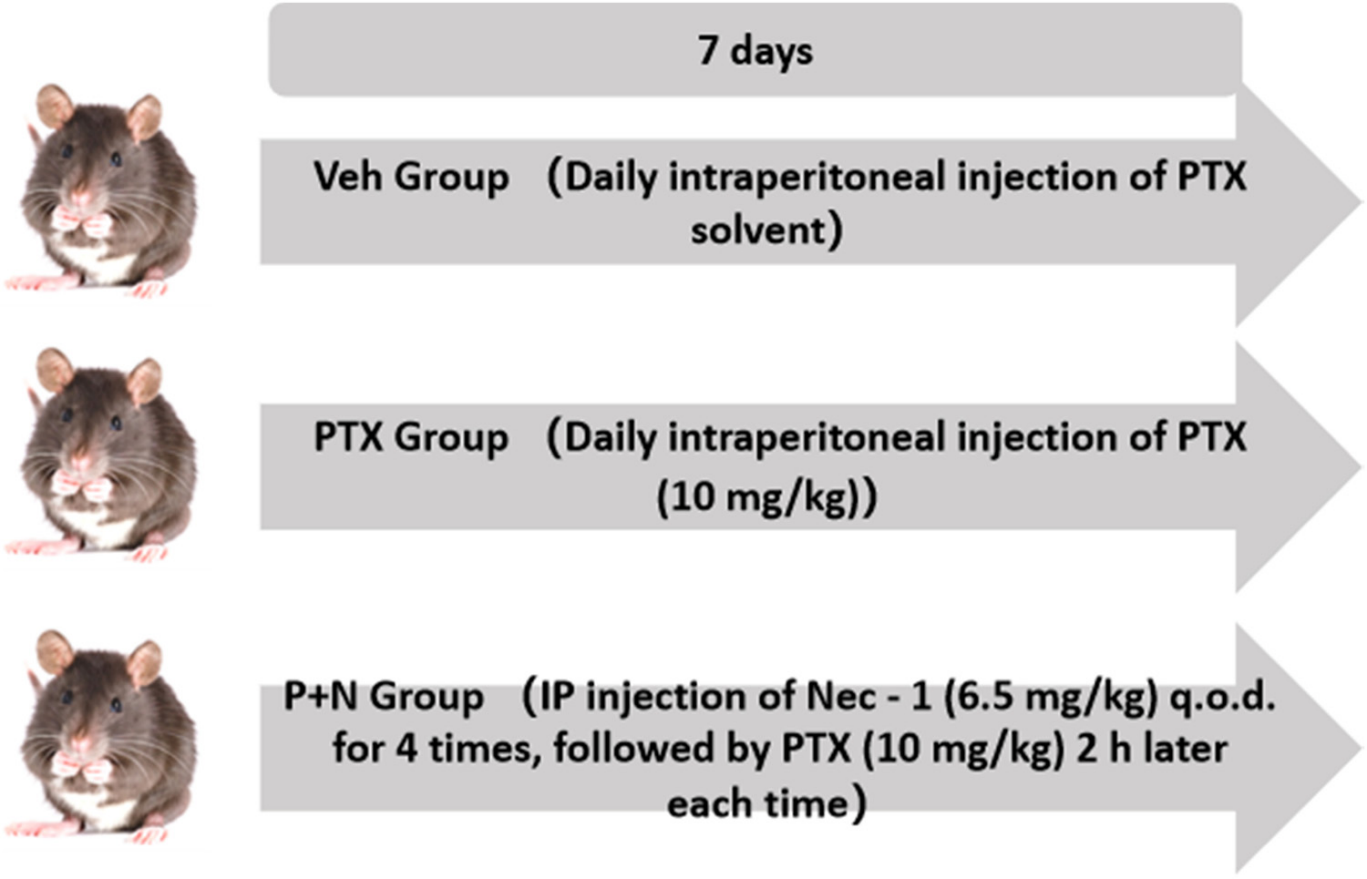
Workflow chart of PTX-induced cognitive impairment mice model construction and group treatment.

1. Veh group (vehicle control)
  As shown in Figure 1, this group received daily intraperitoneal injections of PTX solvent for 7 days and intermittent (every other day) intraperitoneal injections of Nec-1 solvent 2 h before each PTX solvent injection, with a total of four Nec-1 solvent injections.
2. PTX group (PTX only)
  Mice in this group were given daily intraperitoneal injections of PTX (10 mg/kg) for 7 days, along with the same Nec-1 solvent treatment as the Veh group before each PTX injection, as depicted in Figure 1.
3. P + N group (PTX with Nec-1 treatment)
  In this group, as per Figure 1, Nec-1 (6.5 mg/kg) was injected intraperitoneally every other day for four doses, and PTX (10 mg/kg) was injected 2 h after each Nec-1 injection, following the same injection schedule for PTX as the PTX group.

Mice in this group received intraperitoneal injections of PTX solvent (a 1:1 mixture of anhydrous ethanol and Cremophor EL diluted in normal saline) once daily for 7 days. Two hours prior to each PTX solvent injection, mice were intraperitoneally administered an equal dose of Nec-1 solvent (DMSO diluted in normal saline) every other day, for a total of four injections.

1. PTX group (PTX only)
  Mice in the PTX group received intraperitoneal injections of PTX (10 mg/kg; TCI, P1632) once daily for 7 days. Two hours before each PTX injection, they were administered an equal volume of Nec-1 solvent in the same manner as the Veh group.
2. P + N group (PTX with Nec-1 treatment)
  Mice in this group were treated with Nec-1 (6.5 mg/kg; Cayman, 4311-88-0) via intraperitoneal injection every other day for a total of four doses. PTX (10 mg/kg) was injected 2 h after each Nec-1 administration, following the same schedule as the PTX group.

### 2.3 Monitoring and tissue collection

The weight of the mice was recorded daily before drug administration, and their physical condition was closely monitored before and after injections. After successfully establishing the cognitive impairment model, the mice were anesthetized by inhalation of 4–5% isoflurane in pure O₂ at a flow rate of 200–400 mL/min for 3–4 min. Following anesthesia, the mice were euthanized by decapitation, and their chest cavities were opened for tissue collection.

### 2.4 Serum drug concentration analysis

To validate the bioavailability of administered drugs, blood samples were collected from the retro-orbital plexus 1 h after the final PTX or Nec-1 injection. Serum was separated by centrifugation (3,000 rpm, 15 min) and stored at −80°C. Nec-1 and PTX concentrations were quantified using high-performance liquid chromatography (HPLC; Agilent 1260 Infinity II) with a C18 column (4.6 × 150 mm, 5 μm). The mobile phase consisted of acetonitrile:water (70:30 v/v) for Nec-1 and methanol:0.1% formic acid (65:35 v/v) for PTX, at a flow rate of 1.0 mL/min. Detection wavelengths were set at 254 nm (Nec-1) and 227 nm (PTX).

### 2.5 Immunofluorescence staining

Three mice were selected from each group for immunofluorescence staining of brain tissue. After the brain tissue samples were obtained, they were fixed in 4% paraformaldehyde, followed by gradient dehydration in sucrose solutions of different concentrations to ensure that the tissue was better preserved. After dehydration was completed, the tissues were embedded in OCT embedding agent and cut into slices 10 μm thick by microtome.

After antigen repair, the sections were closed with 10% donkey serum at room temperature for 30 min to reduce nonspecific binding. The sections were then incubated overnight at 4°C with primary antibodies against the following targets: Iba-1 (1:200, GeneTex, GTX 632426) for labeling microglia; iNOS (1:200, Arigo, ARG 56509), as markers of M1 microglia; Arg-1 (1:500, BD, 610708) was an important marker of M2 microglia; BDNF (1:500, Abcam, ab108319) for the study of neuroprotective mechanisms; TLR4 (1:100, Affinity, AF7017) and MyD88 (1:100, Affinity, AF5195) are closely related to the signaling pathways of concern in this study.

On the next day, the slices were incubated with secondary antibody for 50 min at room temperature in the dark. Finally, cell nuclei were re-stained with DAPI solution (Shanghai Shengong Biotechnology Co., LTD, E607303) and DAPI fluoresces blue. Fluorescence microscopy was used to examine and collect images. Depending on the fluorescent labels used, the positive cells showed green or red fluorescence, which was convenient for observing and analyzing the expression of different cell markers.

After antigen retrieval, the sections were blocked with 10% donkey serum for 30 min at room temperature. Subsequently, the slices were incubated overnight at 4°C with primary antibodies against the following targets: Iba-1 (1:200, GeneTex, GTX 632426), iNOS (1:200, Arigo, ARG 56509), Arg-1 (1:500, BD, 610708), BDNF (1:500, Abcam, ab108319), TLR4 (1:100, Affinity, AF7017), and MyD88 (1:100, Affinity, AF5195).

The next day, the sections were incubated with secondary antibodies in the dark at room temperature for 50 min. Cell nuclei were counterstained with DAPI solution (Shanghai Sangon Biotechnology Co., Ltd, E607303), which fluoresces blue. Microscopic examination and image acquisition were performed using fluorescence microscopy. Positive cells were visualized in green or red, depending on the fluorescent marker used.

### 2.6 Preparation of conditioned medium for PTX-induced necroptosis of HT22 cells

HT22 cells are widely used to study neuronal cell death mechanisms, including necroptosis. They provide a controlled environment to investigate the effects of PTX on neuronal necroptosis and the subsequent release of DAMPs. HT22 cells (Bluefbio Biology Technology Development Co., Ltd, cat. no. BNF60808571) were seeded into six-well plates following the protocol from our previous study. When the cells reached 70–80% confluence, 10 μM PTX was added to induce necroptosis. For conditioned medium preparation with necroptosis inhibition, 80 μM Nec-1 was added to the culture 2 h before PTX treatment. The detailed procedure is illustrated in Figure 4(a).

**Figure 2:**
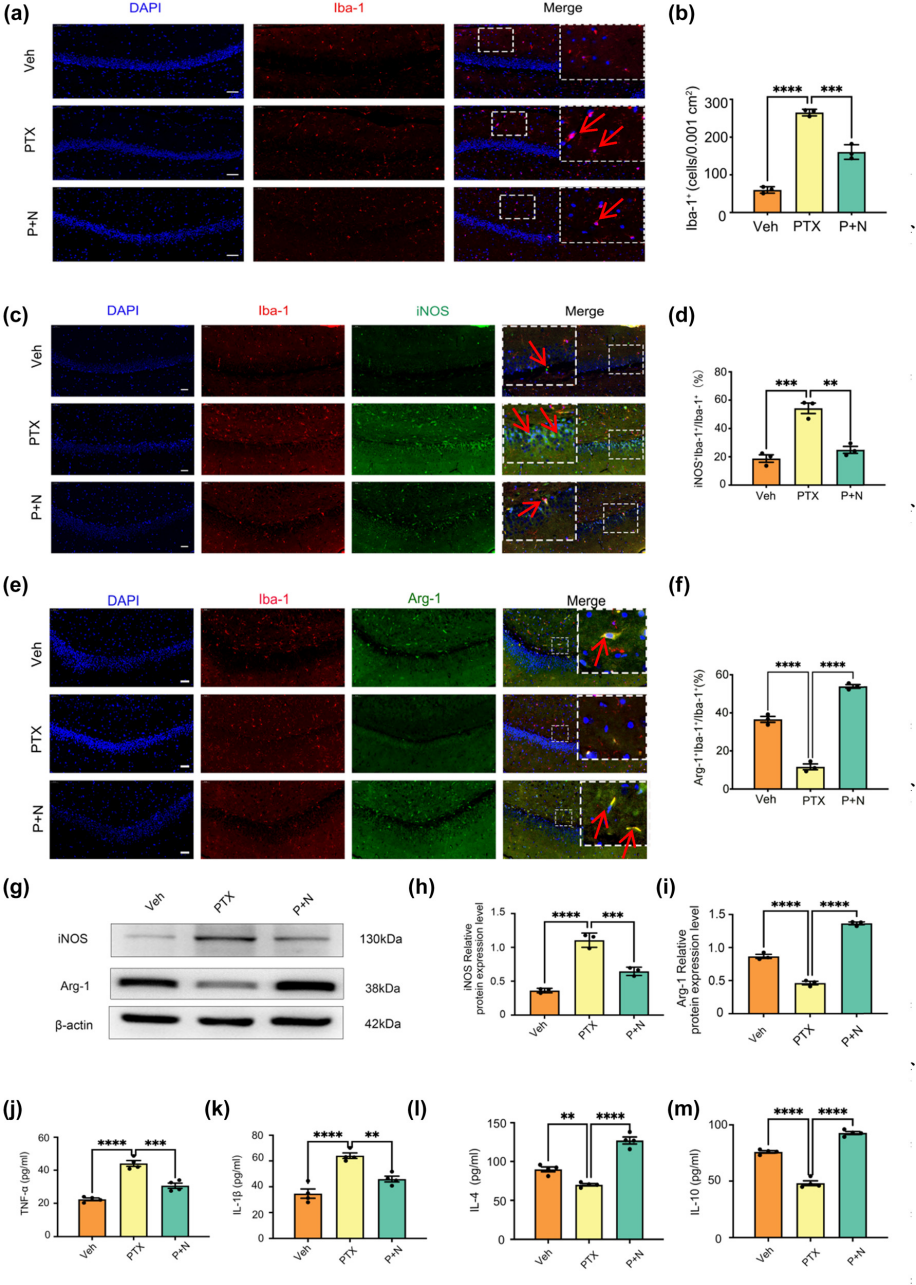
Necroptosis of hippocampal neurons induces microglial polarization toward M1 and inhibits its polarization toward M2. (a) Microphotographs of fluorescent immunohistochemical staining showing labeling of Iba-1 (red). Bar = 50 µm, *n* = 3 mice/group. (b) Number of microglial cells. Results were expressed as the Mean ± SEM, *n* = 3 mice/group, ***P < 0.001, ****P < 0.0001. Abbreviation: Veh, vehicle; PTX, paclitaxel; P + N, paclitaxel + necrostatin-1. (c) Microphotographs of fluorescent immunohistochemical staining showing double labeling of iNOS (green) and Iba-1 (red). Bar = 50 µm. (d) Number of microglial cells and iNOS co-labeled cells as a percentage of the number of microglial cells. Results were expressed as the Mean ± SEM, *n* = 3 mice/group. (e) Microphotographs of fluorescent immunohistochemical staining showing double labeling of Arg-1 (green) and Iba-1 (red). Bar = 50 µm. (f) Number of microglial cells and Arg-1 co-labeled cells as a percentage of the number of microglial cells. Results were expressed as the Mean ± SEM, *n* = 3 mice/group. (g)–(i) Western blot expression of iNOS and Arg-1 in the hippocampus of mice in different groups. (j) and (k) Effects on the levels of inflammatory molecules TNF-α and IL-1β secreted by M1 microglia in the hippocampus of mice in different groups. All data are shown as the Mean ± SEM, *n* = 3 for each group. (l) and (m) Effects on the levels of anti-inflammatory molecules IL-4 and IL-10 secreted by M2 microglia in the hippocampus of mice in different groups. All data are shown as the Mean ± SEM, *n* = 3 for each group.

After 24 h of incubation, the cell culture medium was collected into 1.5 mL centrifuge tubes and centrifuged at 5,000 rpm for 10 min at 4°C. The supernatant was carefully transferred to new centrifuge tubes and stored at −20°C for subsequent experiments.

### 2.7 Culture and treatment of BV-2 cells

BV-2 cells are commonly used to study microglial activation and polarization into pro-inflammatory (M1) and anti-inflammatory (M2) phenotypes. This makes them suitable for investigating how DAMPs released from necroptotic HT22 cells influence microglial behavior. BV-2 cells (Punosai, Cat No.: CL-0493) were revived, passaged, and cultured following the manufacturer’s instructions. The cells were maintained in DMEM supplemented with 10% fetal bovine serum, 1% chloramphenicol, and amphotericin B antibiotics. The incubation conditions were set at 37°C with 5% CO₂ and 95% air.

To investigate the effect of HT22 cell necroptosis-conditioned medium on the polarization of BV-2 cells toward the M1 phenotype, the prepared HT22 necroptosis-conditioned media were added to BV-2 cells. Control groups were treated with PTX at a final concentration of 10 μM or Nec-1 at a final concentration of 80 μM, as appropriate. To further explore the involvement of the TLR4/MyD88 signaling pathway in BV-2 cell polarization, TAK-242 (MCE, Cat No.: 243984-11-4), a TLR4 inhibitor, was added to the cells at a final concentration of 10 μM. TAK-242 was applied 2 h before the addition of the HT22 necroptosis-conditioned medium to block TLR4 signaling [36].

### 2.8 Western blot

Hippocampal tissues and BV-2 cells were homogenized in RIPA lysis buffer (Shanghai Yazyme Biopharmaceutical Technology Co., Ltd, PC101) to extract proteins. Protein samples were separated by SDS-PAGE and transferred onto PVDF membranes (Millipore, IPFL00010).

The membranes were incubated overnight at 4°C with primary antibodies targeting the following proteins: rabbit anti-iNOS (1:1,000, Abcam; ab178945), rabbit anti-Arg-1 (1:1,000, Abcam; ab133543), rabbit anti-BDNF (1:1,000, Abcam; ab108319), rabbit anti-TLR4 (1:500, Affinity; AF7017), rabbit anti-MyD88 (1:500, Affinity; AF5195), and mouse anti-β-actin (1:10,000, Proteintech; 20536-1-AP).

The next day, membranes were incubated with secondary antibodies (anti-rabbit or anti-mouse IgG; 1:5,000, Proteintech, SA00001-2) at room temperature for 1 h. After incubation, ECL luminescent solution (ThermoFisher, 34579) was applied, and immunoblot bands were visualized using the Bio-Rad fluorescence scanning system.

### 2.9 Enzyme-linked immunosorbent assay (ELISA)

The supernatants of hippocampal tissue homogenates and BV-2 cell cultures were collected into EP tubes. The concentrations of TNF-α (Invitrogen, 88-7013), IL-1β (Invitrogen, 88-7324), IL-4 (ESTABIO, CSB-E04634m), and IL-10 (MULTI SCIENCE, EK 210/4-03) in the supernatants were measured using corresponding ELISA kits, following the manufacturers’ protocols. Absorbance at 450 nm was detected using a multi-mode microplate reader (BioTek, Epoch, USA).

### 2.10 Statistical analysis

Statistical analysis was performed using SPSS 26. All data were expressed as the mean ± SEM. Group differences were analyzed using one-way ANOVA followed by the least significant difference *post hoc* test. For data with heterogeneous variance or non-normal distributions, the Kruskal–Wallis *H* test was applied. A *P*-value of <0.05 was considered statistically significant.


**Ethics approval:** The experimental procedures in this study were approved by the Experimental Animal Welfare Ethics Committee of Hebei Medical University (Approval No. IACUC-Hebmu-2020009).

## 3 Results

### 3.1 Validation of serum drug levels

HPLC analysis confirmed that serum Nec-1 and PTX concentrations reached 12.3 ± 1.5 and 8.7 ± 0.9 μM, respectively, 1 h post-injection. These levels are consistent with previous reports demonstrating effective RIPK1 inhibition by Nec-1 and neurotoxic effects of PTX in rodents, supporting the appropriateness of the selected doses.

### 3.2 PTX-induced cognitive impairment in mice increases microglial activation in the hippocampus

Previous studies by our team demonstrated that PTX-induced cognitive impairment is closely associated with the necroptosis of hippocampal neurons [18]. These necroptotic neurons release large amounts of DAMPs [7]. As endogenous danger signals, DAMPs are released following tissue damage and can trigger the infiltration of various immune cells, including microglia [8]. We hypothesize that PTX-induced DAMP released in the hippocampus may lead to microglial infiltration, subsequently triggering a severe neuroinflammatory response.

To investigate microglial activation, we focused on Iba-1, a calcium-binding protein and specific marker of microglia. Iba-1 expression increases during microglial activation, transitioning microglia from a quiescent to an active state in response to nervous system damage. Immunofluorescence staining was employed to examine the distribution and expression of microglia in the hippocampus. We further quantified the morphological differences of microglia in the three groups.

For the branch ratio of microglia, we defined the branch ratio as the number of primary branches divided by the total number of visible processes of each microglia. In the Veh group, the average branch ratio of Iba-1-positive microglia was 0.65 ± 0.05 (Figure 2(a) and (b)). In the PTX group, the branch ratio significantly decreased to 0.35 ± 0.04 (*P* < 0.05 compared to the Veh group), consistent with the observed “moth-eaten” distribution and reduced branching. In the P + N group, the branch ratio increased to 0.55 ± 0.05, which was significantly higher than that in the PTX group (*P* < 0.05) and indicated a partial restoration of the branched morphology.

**Figure 3:**
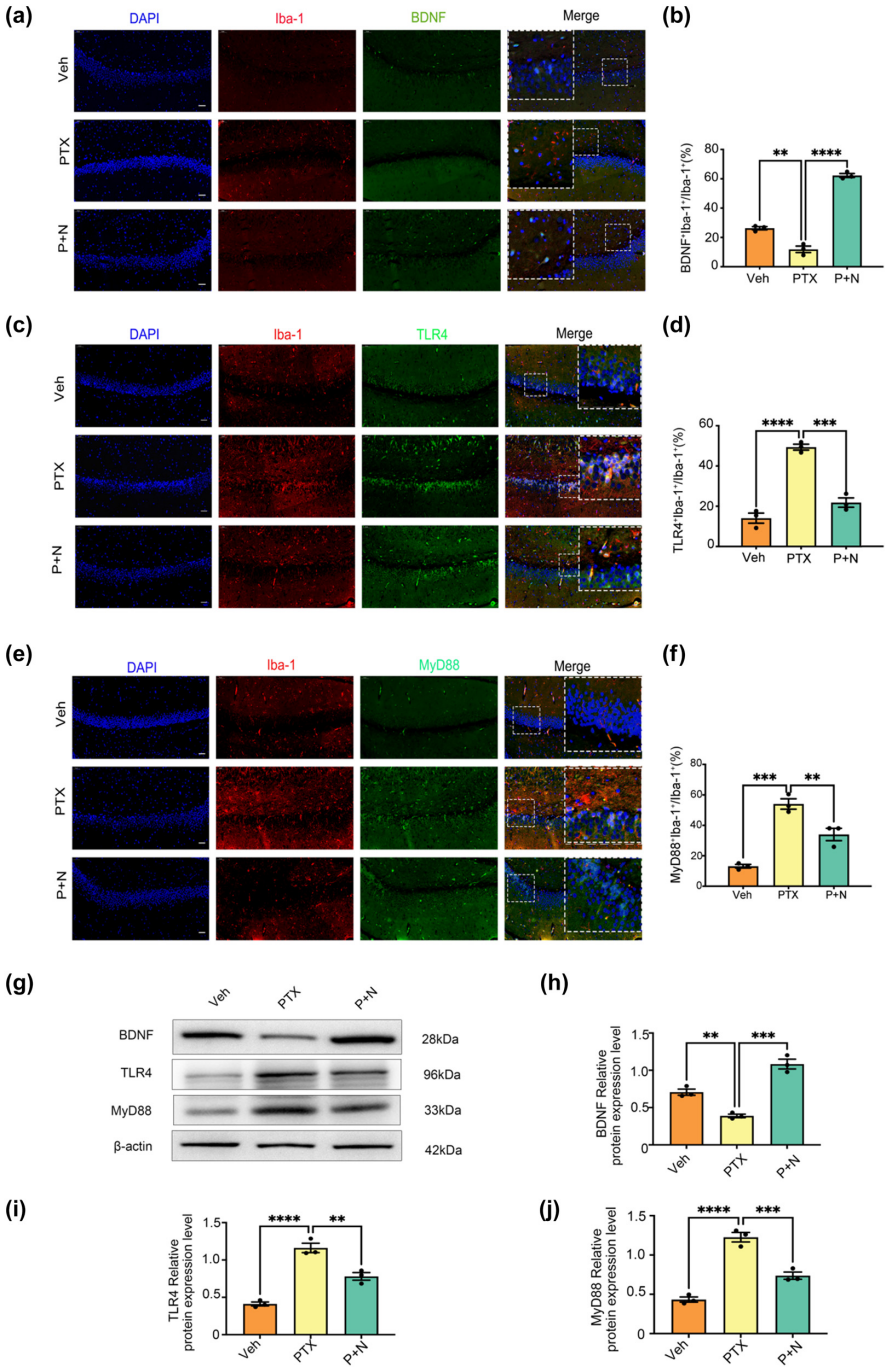
Necroptosis of hippocampal neurons leads to decreased BDNF release from microglia and activation of TLR4/MyD88 signaling pathway. (a) Microphotographs of fluorescent immunohistochemical staining showing double labeling of BDNF (green) and Iba-1 (red). Bar = 50 µm. (b) Number of microglial cells and BDNF co-labeled cells as a percentage of the number of microglial cells. Results were expressed as the Mean ± SEM, *n* = 3 mice/group, **P < 0.01, ****P < 0.0001. Abbreviation: Veh, vehicle; PTX, paclitaxel; P + N, paclitaxel + necrostatin-1. (c) Microphotographs of fluorescent immunohistochemical staining showing double labeling of TLR4 (green) and Iba-1 (red). Bar = 50 µm. (d) Number of microglial cells and TLR4 co-labeled cells as a percentage of the number of microglial cells. Results were expressed as the Mean ± SEM, *n* = 3 mice/group. (e) Microphotographs of fluorescent immunohistochemical staining showing double labeling of MyD88 (green) and Iba-1 (red). Bar = 50 µm. (f) Number of microglial cells and MyD88 co-labeled cells as a percentage of the number of microglial cells. Results were expressed as the Mean ± SEM, *n* = 3 mice/group. (g)–(j) Western blot expression of BDNF, TLR4, and MyD88 in the hippocampus of mice in different groups. All data are shown as the Mean ± SEM, *n* = 3 for each group.

Regarding the count of microglia, in the Veh group, the number of Iba-1-positive microglia per high-power field (HPF) was 25 ± 3. The PTX group showed a significant increase, with 45 ± 4 microglia per HPF (*P* < 0.05 compared to the Veh group). In the P + N group, the number of Iba-1-positive microglia per HPF decreased to 30 ± 3, which was significantly lower than that in the PTX group (*P* < 0.05, Figure 2(a) and (b)). These results suggest that PTX-induced cognitive impairment causes significant changes in microglial activation and morphology. Observing these morphological differences through immunofluorescence, we further speculated that microglia in each group may exist in different polarization states.

### 3.3 PTX-induced hippocampal neuron necroptosis drives M1 microglial polarization, while its inhibition promotes M2 polarization

To investigate the effect of PTX-induced hippocampal neuron necroptosis on microglial polarization, we examined the expression of inducible nitric oxide synthase (iNOS) as an M1 marker and arginase-1 (Arg-1) as an M2 marker. Double immunofluorescence staining revealed a significant increase in (Iba-1 + iNOS +) M1 microglia in the PTX group compared to the Veh group (*P* < 0.05, Cohen’s *d* = 1.2, 95% CI [0.8, 1.6], Figure 2(c) and (d)), while the number of (Iba-1 + Arg-1 +) M2 microglia significantly decreased (*P* < 0.05, Cohen’s *d* = −1.3, 95% CI [−1.7, −0.9], Figure 2(e) and (f)). In the P + N group, the number of (Iba-1 + iNOS +) cells was significantly reduced compared to the PTX group (*P* < 0.05, Cohen’s *d* = −1.1, 95% CI [−1.5, −0.7], Figure 2(c) and (d)), whereas the number of (Iba-1+ Arg-1+) M2 microglia was significantly increased (P < 0.05, Cohen’s *d* = 1.4, 95% CI [1.0, 1.8], Figure 2(e) and (f)).

Western blot results further corroborated these findings. In hippocampal tissues, the PTX group exhibited significantly higher iNOS expression compared to the Veh group (*P* < 0.05, Cohen’s *d* = 1.5, 95% CI [1.1, 1.9], Figure 2(g) and (h)) and significantly lower Arg-1 expression (*P* < 0.05, Cohen’s *d* = −1.4, 95% CI [−1.8, −1.0], Figure 2(g) and (i)). Treatment with Nec-1 in the P + N group markedly reduced iNOS expression (*P* < 0.05, Cohen’s *d* = −1.3, 95% CI [−1.7, −0.9], Figure 2(g) and (h)) while significantly increasing Arg-1 expression (*P* < 0.05, Cohen’s *d* = 1.6, 95% CI [1.2, 2.0], Figure 2(g) and (i)).

ELISA results showed that PTX treatment led to significantly elevated levels of the pro-inflammatory cytokines TNF-α and IL-1β compared to the Veh group (*P* < 0.05, TNF-α: Cohen’s *d* = 1.6, 95% CI [1.2, 2.0]; IL-1β: Cohen’s *d* = 1.5, 95% CI [1.1, 1.9], Figure 2(j) and (k)), while levels of the anti-inflammatory cytokines IL-4 and IL-10 were significantly reduced (*P* < 0.05, IL-4: Cohen’s *d* = −1.4, 95% CI [−1.8, −1.0]; IL-10: Cohen’s *d* = −1.3, 95% CI [−1.7, −0.9], Figure 2(l) and (m)). In the P + N group, TNF-α and IL-1β levels were significantly reduced compared to the PTX group (*P* < 0.05, TNF-α: Cohen’s *d* = −1.4, 95% CI [−1.8, −1.0]; IL-1β: Cohen’s *d* = −1.3, 95% CI [−1.7, −0.9], Figure 2(j) and (k)), whereas IL-4 and IL-10 levels were significantly increased (*P* < 0.05, IL-4: Cohen’s *d* = 1.5, 95% CI [1.1, 1.9]; IL-10: Cohen’s *d* = 1.4, 95% CI [1.0, 1.8], Figure 2(l) and (m)). These findings demonstrate that PTX-induced cognitive impairment is associated with hippocampal neuron necroptosis, which drives microglial polarization toward the M1 pro-inflammatory phenotype. In contrast, inhibiting hippocampal neuron necroptosis promotes M2 anti-inflammatory microglial polarization and reduces neuroinflammatory responses (as shown in Figure 2 and related data). Although the current data in Figure 2 do not directly support the improvement of cognitive function by inhibiting microglial polarization, based on previous studies showing the neuroprotective role of M2 microglia, we hypothesize that this shift in microglial polarization may contribute to cognitive function improvement.

### 3.4 Effect of hippocampal neuron necroptosis on BDNF expression in PTX-induced cognitive impairment

To explore the neuroprotective role of M2 microglia, we used double immunofluorescence staining for BDNF and Iba-1 in hippocampal tissue. In the PTX group, BDNF-positive cells were significantly reduced, with few co-labeled with Iba-1. In contrast, the P + N group had a significant increase in (Iba-1 + BDNF +) cells (*P* < 0.05, Cohen’s *d* = 1.3, 95% CI [0.9, 1.7], Figure 3(a) and (b)). This large effect size (Cohen’s *d* = 1.3) indicates a substantial difference between the groups, and the 95% CI [0.9, 1.7] shows the reliability of this difference.

**Figure 4:**
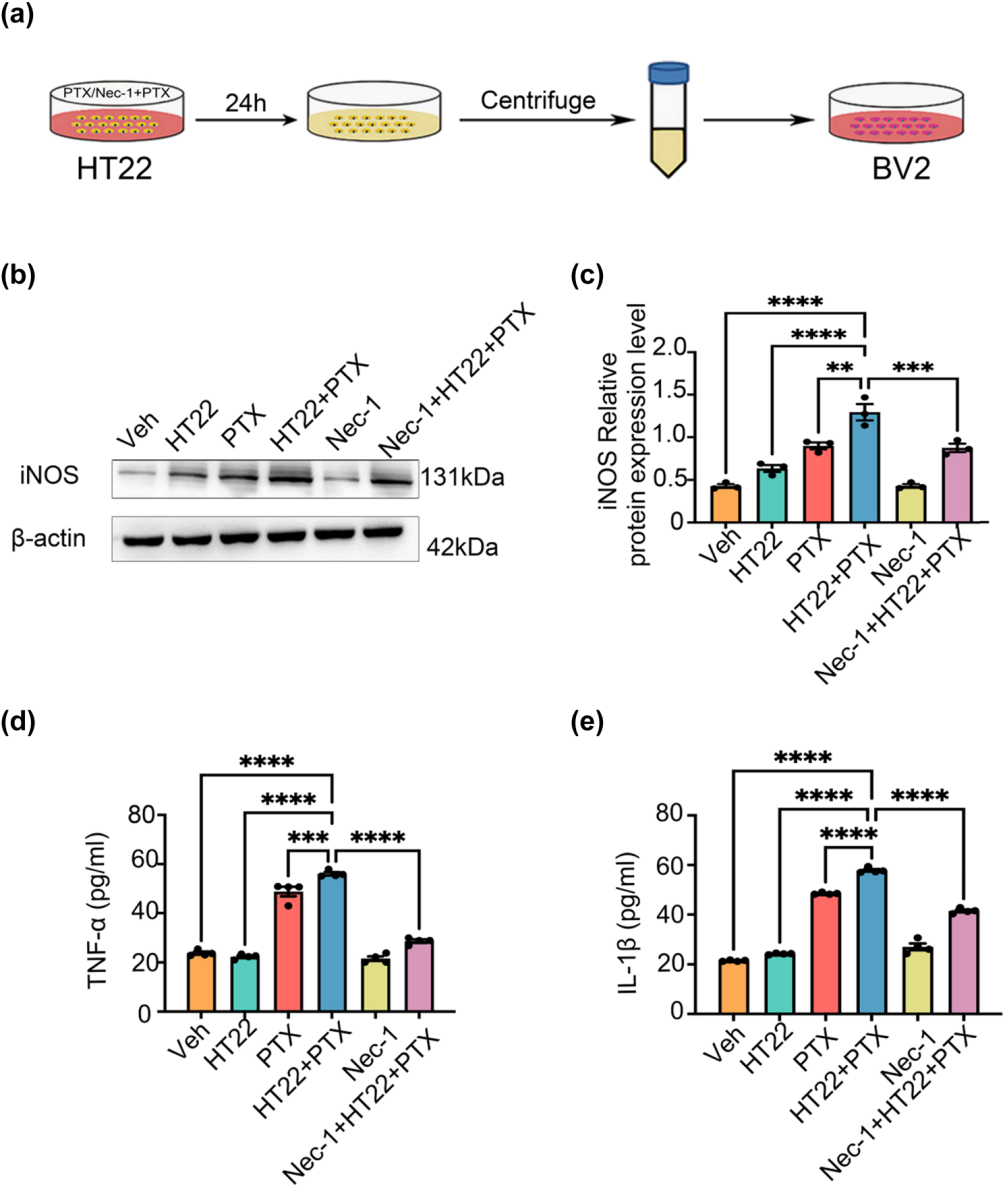
Preparation of DAMPs in the conditioned medium for PTX-induced necroptosis of HT22 cells and the conditioned medium for PTX-induced necroptosis of HT22 cells can induce BV-2 cells to polarize toward M1. (a) Preparation of PTX-induced necroptosis-conditioned culture medium of HT22 cells. (b) and (c) Effects of necroptosis-conditioned culture medium of different groups of HT22 cells on iNOS expression in BV-2 cells. (d) Effects of necroptosis-conditioned culture medium of different groups of HT22 cells on TNF-α release of BV-2 cells. (e) Effects of necroptosis-conditioned culture medium of different groups of HT22 cells on IL-1β release of BV-2 cells. All data are shown as the Mean ± SEM, *n* = 3 mice/group, **P < 0.01, ***P < 0.001, ****P < 0.0001. Abbreviation: Veh, vehicle; PTX, paclitaxel; P + N, paclitaxel + necrostatin-1.

Western blot analysis further supported these results. BDNF expression was significantly higher in the P + N group than in the PTX group (*P* < 0.05, Cohen’s *d* = 1.4, 95% CI [1.0, 1.8], Figure 3(g) and (h)). The effect size of 1.4 implies a large practical difference, and the 95% CI [1.0, 1.8] indicates high precision. In summary, inhibiting hippocampal neuron necroptosis increases M2 microglia, which may promote neuronal survival and repair by secreting higher levels of BDNF. This mechanism may partially explain the cognitive improvements observed after inhibiting hippocampal neuron necroptosis.

### 3.5 Role of TLR4/MyD88 signaling pathway in microglial polarization induced by hippocampal neuron necroptosis

TLRs are conserved proteins that regulate immune responses and detect cell damage. Among them, TLR4 recognizes both exogenous lipopolysaccharide (LPS) from Gram-negative bacteria and endogenous molecules released by necrotic cells, such as heat shock proteins and degraded enzymes [24]. Thus, TLR4 can detect both pathogens and endogenous danger signals. Prior studies have shown that TLR4 is involved in necroptosis across various diseases [37,38]. Based on these findings, this study investigated the role of TLR4 in PTX-induced cognitive impairment and the effect of inhibiting hippocampal neuron necroptosis on TLR4 expression.

Double immunofluorescence revealed a significant increase in TLR4-positive cells in the PTX group compared to the Veh group, with most TLR4-positive cells co-labeled with Iba-1 (*P* < 0.05, Cohen’s *d* = 1.5, 95% CI [1.1, 1.9], Figure 3(c) and (d)). This large effect size (Cohen’s *d* = 1.5) indicates a substantial difference between the two groups, suggesting that PTX treatment leads to a notable increase in TLR4-expressing microglia. The 95% CI [1.1, 1.9] implies a high level of confidence in the reliability of this difference. In the P + N group, the number of TLR4 and Iba-1 co-labeled cells was significantly reduced compared to the PTX group (*P* < 0.05, Cohen’s *d* = −1.2, 95% CI [−1.6, −0.8], Figure 3(c) and (d)). The negative Cohen’s *d* value of −1.2 shows a reverse and substantial difference, meaning that inhibiting necroptosis in the P + N group effectively reduces the number of TLR4-expressing microglia. The CI [−1.6, −0.8] further validates the reliability of this reduction.

Western blot analysis confirmed these results, showing significantly higher TLR4 protein expression in the hippocampal tissues of the PTX group compared to the Veh group (*P* < 0.05, Cohen’s *d* = 1.6, 95% CI [1.2, 2.0], Figure 3(g) and (i)). This indicates a large and reliable difference in TLR4 protein levels between the two groups. In the P + N group, TLR4 expression was significantly reduced compared to the PTX group (*P* < 0.05, Cohen’s *d* = −1.3, 95% CI [−1.7, −0.9], Figure 3(g) and (i)), again demonstrating a substantial and reliable decrease in TLR4 expression when necroptosis is inhibited.

MyD88, an intracellular adapter molecule, plays a critical role in the downstream signaling of TLR4 [39]. To further investigate TLR4 pathway activation, we analyzed MyD88 expression and distribution in hippocampal tissues. Double immunofluorescence showed a significant increase in MyD88-positive cells in the PTX group compared to the Veh group, with most cells co-labeled with Iba-1 (*P* < 0.05, Cohen’s *d* = 1.4, 95% CI [1.0, 1.8], Figure 3(e) and (f)). The effect size of 1.4 indicates a large difference, suggesting that PTX treatment activates the TLR4/MyD88 pathway, leading to an increase in MyD88-expressing microglia. However, the number of MyD88 and Iba-1 co-labeled cells was significantly reduced in the P + N group (*P* < 0.05, Cohen’s *d* = −1.1, 95% CI [−1.5, −0.7], Figure 3(e) and (f)), showing that inhibiting necroptosis reduces MyD88-expressing microglia.

Western blot analysis further confirmed these findings, showing significantly higher MyD88 protein expression in the PTX group compared to the Veh group (*P* < 0.05, Cohen’s *d* = 1.5, 95% CI [1.1, 1.9], Figure 3(g) and (j)). In the P + N group, MyD88 protein expression was significantly decreased compared to the PTX group (*P* < 0.05, Cohen’s *d* = −1.2, 95% CI [−1.6, −0.8], Figure 3(g) and (j)). These results were consistent with the immunofluorescence data, providing strong evidence that the TLR4/MyD88 signaling pathway is involved in the microglial polarization induced by hippocampal neuron necroptosis in PTX-induced cognitive impairment.

### 3.6 HT22 cell necroptosis-conditioned medium induces M1 polarization of BV-2 cells

To investigate whether PTX-induced hippocampal neuron necroptosis regulates microglial polarization, we treated BV-2 microglia with conditioned medium from HT22 cells and analyzed the expression of M1 polarization markers, including iNOS and inflammatory factors. As shown in Figure 4(a) and (b) V-2 cells were treated with different types of HT22 cell-conditioned medium to evaluate their polarization state.

Compared to the Veh group, conditioned medium from PTX-induced necroptotic HT22 cells significantly promoted BV-2 cell polarization toward the M1 phenotype, as evidenced by a significant increase in iNOS expression (*P* < 0.05, Cohen’s *d* = 1.4, 95% CI [1.0, 1.8], Figure 4(b) and (c)). The large effect size indicates a substantial increase in iNOS expression, suggesting a strong induction of M1 polarization. Additionally, the levels of pro-inflammatory factors TNF-α and IL-1β were markedly elevated (*P* < 0.05, TNF-α: Cohen’s *d* = 1.5, 95% CI [1.1, 1.9]; IL-1β: Cohen’s *d* = 1.6, 95% CI [1.2, 2.0], Figure 4(d) and (e)). These effect sizes further confirm the induction of an inflammatory response associated with M1 polarization.

Conversely, when HT22 cells were pretreated with Nec-1 to inhibit necroptosis, iNOS expression (*P* < 0.05, Cohen’s *d* = −1.3, 95% CI [−1.7, −0.9], Figure 4(b) and (c)) and the levels of TNF-α and IL-1β in the conditioned medium were significantly reduced (*P* < 0.05, TNF-α: Cohen’s *d* = −1.4, 95% CI [−1.8, −1.0]; IL-1β: Cohen’s *d* = −1.5, 95% CI [−1.9, −1.1], Figure 4(d) and (e)). The negative effect sizes indicate that inhibiting necroptosis effectively reduces M1 polarization and the associated inflammatory response.

Both western blot and ELISA results confirmed that conditioned medium from PTX-induced necroptotic HT22 cells effectively drives BV-2 cells toward M1 polarization. Importantly, inhibiting neuronal necroptosis using Nec-1 significantly reduced M1 polarization of microglia and the release of inflammatory factors. These *in vitro* findings align closely with the *in vivo* results.

### 3.7 Effect of TAK-242 on M1/M2 polarization of BV-2 cells induced by HT22 cell necroptosis-conditioned medium

To further explore the involvement of the TLR4/MyD88 signaling pathway in microglial polarization induced by hippocampal neuron necroptosis, we used the TLR4-specific blocker TAK-242 in *in vitro* experiments [36]. Three experimental groups were established: (1) Veh group (solvent control), (2) DAMPs group (HT22 necroptosis-conditioned medium), and (3) DAMPs + TAK-242 group (HT22 necroptosis-conditioned medium with TAK-242).

**Figure 5:**
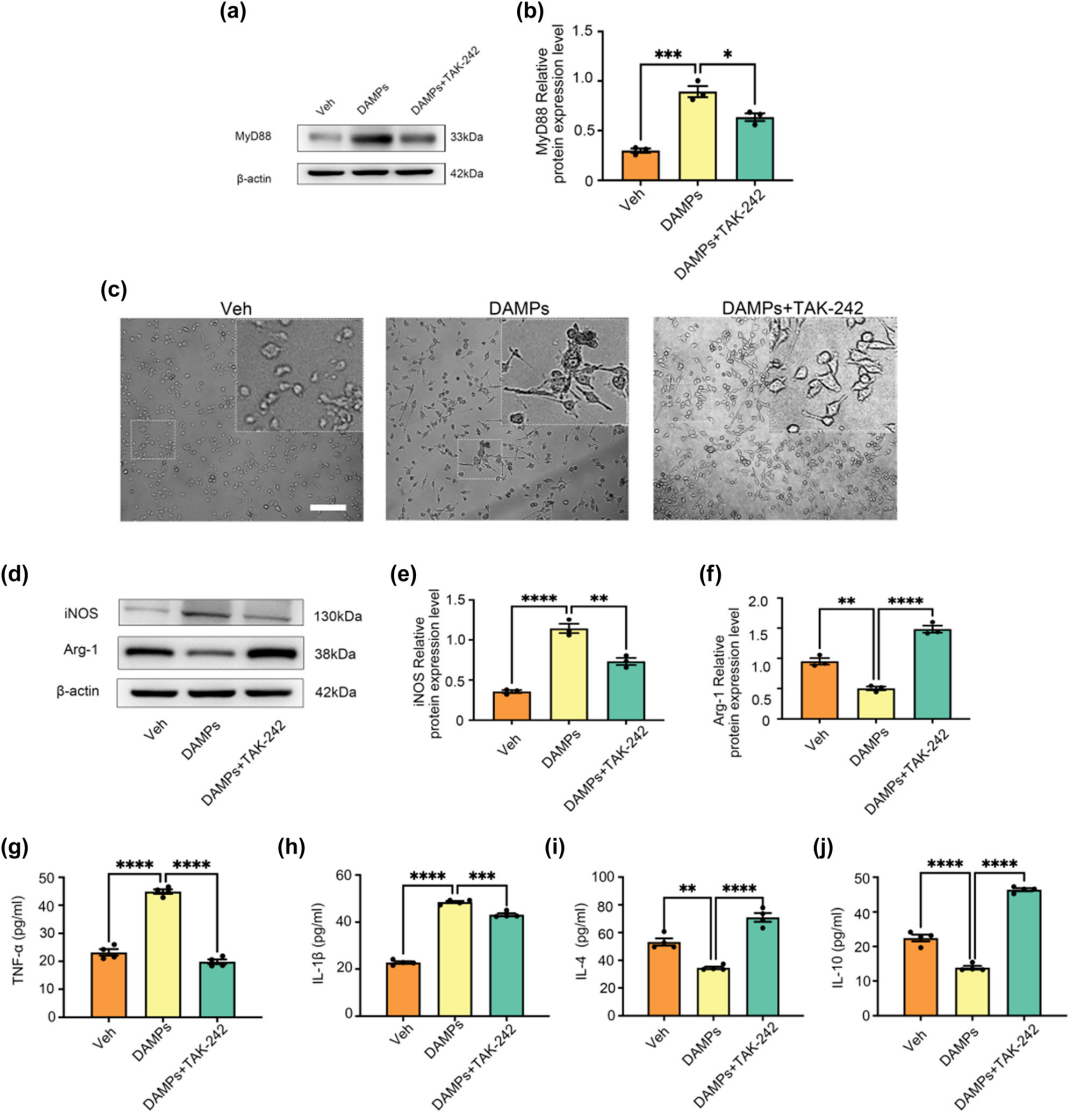
Polarization of microglia induced by conditioned medium of HT22 cells necroptosis is mediated by TLR4/MyD88 signaling pathway. (a) and (b) Effect of TAK-242 on MyD88 protein expression in BV-2 cells caused by necroptosis of HT22 cells in conditioned culture medium. All data are shown as the Mean ± SEM, *n* = 3, *P < 0.05, **P < 0.01, ***P < 0.001, ****P < 0.0001. Abbreviation: Veh, vehicle; PTX, paclitaxel; P + N, paclitaxel + necrostatin-1. (c) Morphological changes of BV-2 cells in different treatment groups. Bar = 100 µm. (d)–(f) Western blot expression of iNOS and Arg-1 in the BV-2 cells in different groups. (g)–(h) Effects on the levels of inflammatory molecules TNF-α and IL-1β secreted by M1 microglia in the BV-2 cells in different groups. (i) and (j) Effects on the levels of anti-inflammatory molecules IL-4 and IL-10 secreted by M2 microglia in the BV-2 cells in different groups. All data are shown as the Mean ± SEM, *n* = 3 for each group.

Compared to the Veh group, the DAMPs group showed significantly increased MyD88 protein expression, consistent with the *in vivo* results (*P* < 0.05, Cohen’s *d* = 1.5, 95% CI [1.1, 1.9]). Pretreatment with TAK-242 in the DAMPs + TAK-242 group markedly reduced MyD88 expression compared to the DAMPs group (*P* < 0.05, Cohen’s *d* = −1.3, 95% CI [−1.7, −0.9], Figure 5(a) and (b)). These effect sizes demonstrate the activation of the TLR4/MyD88 pathway by the DAMPs and the inhibitory effect of TAK-242 on this pathway.

Morphological observations under an inverted phase-contrast microscope revealed that BV-2 cells in the Veh group displayed a resting state, characterized by round or spindle-shaped cells with clear boundaries. In contrast, BV-2 cells in the DAMPs group showed an activated state, with shorter, thicker, amoeba-like shapes and numerous projections. Pretreatment with TAK-242 attenuated these morphological changes, indicating its inhibitory effect on BV-2 cell activation (Figure 5(c)).

Western blot analysis showed significantly increased iNOS expression in the DAMPs group compared to the Veh group (*P* < 0.05, Cohen’s *d* = 1.6, 95% CI [1.2, 2.0], Figure 5(d) and (e)) and significantly decreased Arg-1 expression (*P* < 0.05, Cohen’s *d* = −1.4, 95% CI [−1.8, −1.0], Figure 5(d) and (f)). In the DAMPs + TAK-242 group, iNOS expression was significantly reduced (*P* < 0.05, Cohen’s *d* = −1.4, 95% CI [−1.8, −1.0], Figure 5(d) and (e)), while Arg-1 expression was significantly increased (*P* < 0.05, Cohen’s *d* = 1.5, 95% CI [1.1, 1.9], Figure 5(d) and (f)), suggesting that TAK-242 effectively regulates the transition from M1 to M2 polarization.

ELISA results further confirmed these findings. Compared to the Veh group, the DAMPs group exhibited significantly increased levels of pro-inflammatory cytokines TNF-α and IL-1β (*P* < 0.05, TNF-α: Cohen’s *d* = 1.7, 95% CI [1.3, 2.1]; IL-1β: Cohen’s *d* = 1.6, 95% CI [1.2, 2.0], Figure 5(g) and (h)) and significantly reduced levels of anti-inflammatory cytokines IL-4 and IL-10 (*P* < 0.05, IL-4: Cohen’s *d* = −1.5, 95% CI [−1.9, −1.1]; IL-10: Cohen’s *d* = −1.4, 95% CI [−1.8, −1.0], Figure 5(i) and (j)). In the DAMPs + TAK-242 group, TNF-α and IL-1β levels were significantly reduced (*P* < 0.05, TNF-α: Cohen’s *d* = −1.5, 95% CI [−1.9, −1.1]; IL-1β: Cohen’s *d* = −1.4, 95% CI [−1.8, −1.0], Figure 5(g) and (h)), while IL-4 and IL-10 levels were significantly increased (*P* < 0.05, IL-4: Cohen’s *d* = 1.6, 95% CI [1.2, 2.0]; IL-10: Cohen’s *d* = 1.5, 95% CI [1.1, 1.9], Figure 5(i) and (j)). This study evaluated the role of PTX-induced hippocampal neuron necroptosis in microglial polarization using immunofluorescence, western blot, and ELISA. Additionally, *in vitro* experiments revealed that the TLR4/MyD88 signaling pathway mediates microglial polarization induced by hippocampal neuron necroptosis.

## 4 Discussion

In this study, to explore the underlying mechanism of PTX-induced cognitive impairment, we conducted *in vivo* and *in vitro* experiments. *In vivo*, adult male C57BL/6N mice were divided into Veh, PTX, and P + N groups to establish a PTX-induced cognitive impairment model (Figure 2). We used techniques like immunofluorescence staining, western blot, and ELISA to detect the expression of microglial markers, M1 and M2 phenotype markers, cytokines, BDNF, and proteins related to the TLR4/MyD88 signaling pathway in the hippocampus. *In vitro*, we induced necroptosis in HT22 cells, prepared conditioned medium, and treated BV-2 cells with it. We also added Nec-1 to inhibit HT22 cell necroptosis and TAK-242 to block the TLR4/MyD88 signaling pathway to investigate their effects on BV-2 cell polarization. Overall, our results demonstrated for the first time that hippocampal neuron necroptosis activates microglia through the TLR4/MyD88 signaling pathway in PTX-induced cognitive impairment, promoting M1 polarization and neuroinflammation, while inhibiting necroptosis promotes M2 polarization and neuroprotection.

### 4.1 Microglial polarization and neuroinflammation

Immunofluorescence staining of Iba-1 demonstrated that PTX-induced cognitive impairment caused microglia to adopt an amoeboid morphology, characteristic of M1 polarization, which is consistent with our previous findings [40]. Inhibition of hippocampal neuron necroptosis by Nec-1 significantly reduced Iba-1-positive cells and altered their morphology, suggesting a shift toward the M2 anti-inflammatory phenotype [41]. M1 microglia, marked by iNOS, promotes inflammation through the release of pro-inflammatory cytokines such as TNF-α and IL-1β [42,43]. In contrast, M2 microglia, marked by Arg-1, release anti-inflammatory cytokines such as IL-4 and IL-10, contributing to tissue repair and neuroprotection [44–46]. Our findings showed that Nec-1 treatment significantly reduced iNOS expression while increasing Arg-1 levels in microglia, as well as altering cytokine secretion patterns [47]. These results suggest that inhibiting necroptosis can suppress the pro-inflammatory effects of M1 microglia while promoting the reparative functions of M2 microglia [48]. BDNF, a key neurotrophic factor, is essential for maintaining cognitive function [49–51]. Our findings showed that inhibiting hippocampal neuron necroptosis significantly increased BDNF expression in microglia [52]. Specifically, the number of (Iba-1+ BDNF+) cells increased in the P + N group compared to the PTX group, indicating that M2 microglia secretes higher levels of BDNF. This mechanism may explain the improved cognitive outcomes observed after Nec-1 treatment.

### 4.2 TLR4/MyD88 signaling pathway in microglial polarization

Previous studies have investigated the role of TLR4/MyD88 signaling in neuroinflammation. However, this study uniquely focuses on the mechanism by which hippocampal neuron necroptosis activates microglia through this pathway in the context of PTX-induced cognitive impairment [53]. TLR4 is a critical receptor in innate immunity that recognizes DAMPs released during cell necroptosis and activates downstream inflammatory pathways, including NF-κB and MAPK [54–56]. In this study, PTX-induced hippocampal neuron necroptosis significantly increased the expression of TLR4 and MyD88 in microglia, as demonstrated by immunofluorescence and western blot analysis. Inhibition of necroptosis reduced the expression of both TLR4 and MyD88 [57], suggesting that this pathway mediates microglial polarization during PTX-induced neuroinflammation. This is distinct from previous studies which did not explore the connection between neuron necroptosis and microglial activation via TLR4/MyD88 in the context of PTX-induced cognitive impairment. Additionally, previous work documented PTX-induced M1 polarization of microglia [58]. But this study further explores the regulatory effect of Nec-1 on this process and its underlying mechanism, providing new insights into potential therapeutic strategies [59]. *In vitro* experiments further validated that conditioned medium from PTX-induced necroptotic HT22 cells drives BV-2 microglia toward M1 polarization. Nec-1 treatment reduced this effect, supporting the role of necroptosis in microglial polarization. To explore the involvement of TLR4/MyD88, we used TAK-242, a specific TLR4 inhibitor. TAK-242 treatment effectively reduced MyD88 expression in BV-2 cells and reversed M1 polarization, as indicated by decreased iNOS expression and increased Arg-1 levels [60]. ELISA results showed that TAK-242 also reduced pro-inflammatory cytokines (TNF-α and IL-1β) while increasing anti-inflammatory cytokines (IL-4 and IL-10), confirming its regulatory effect [61].

Although our data show an increase in M2 microglia and BDNF expression after inhibiting hippocampal neuron necroptosis, the direct causal relationship between these changes and cognitive function improvement is yet to be established. In previous studies, BDNF has been shown to play a crucial role in neuronal survival, differentiation, and synaptic plasticity, which are closely related to cognitive function [62]. Our findings of increased BDNF expression in M2 microglia after inhibiting necroptosis suggest a potential mechanism for cognitive improvement. However, to confirm this hypothesis, future studies could use techniques such as BDNF gene knockdown or overexpression in the context of PTX-induced cognitive impairment models. By directly manipulating BDNF levels, we can better understand its role in mediating the effects of inhibiting hippocampal neuron necroptosis on cognitive function. Additionally, more comprehensive behavioral tests, such as the Morris Water Maze and Novel Object Recognition tests, are needed to accurately assess cognitive function changes in relation to these molecular and cellular alterations.

Regarding the potential therapeutic agents Nec-1 and TAK-242 identified in this study, it is crucial to consider their safety profiles for clinical translation. Nec-1, as a RIPK1 inhibitor, has shown promising effects in this study in inhibiting necroptosis and reducing microglial M1 polarization. However, in some previous studies, it has been reported to have potential side effects. For example, high-dose Nec-1 treatment in animal models was associated with mild hepatic and renal function abnormalities, likely due to its interference with normal cellular processes in these organs [53]. Although the doses used in this study did not cause overt systemic toxicity, careful consideration of these potential side effects is necessary when translating to clinical applications.

TAK-242, a TLR4 inhibitor, has also been shown to be effective in modulating microglial polarization in our experiments. But its safety concerns should not be overlooked. Some studies have suggested that long-term use of TAK-242 may disrupt the normal immune response in the body, as TLR4 is an important receptor in the innate immune system. This could potentially increase the risk of infections or interfere with the body’s ability to clear pathogens [58].

Therefore, future research should focus on further evaluating the safety of Nec-1 and TAK-242 in more relevant *in vivo* models, and optimizing treatment regimens to minimize potential side effects while maintaining therapeutic efficacy.

### 4.3 Limitations and future directions

Although our findings demonstrate that TLR4/MyD88 mediates microglial polarization induced by hippocampal neuron necroptosis, this study has several limitations. First, we did not conduct dose–response studies for PTX treatment. The chosen PTX dose (10 mg/kg) was based on previous research, but a lack of dose–response data restricts a more comprehensive understanding of the relationship between PTX dosage and the observed effects. In future research, we plan to set up multiple PTX treatment groups with different doses to investigate the dose-dependent effects on hippocampal neuron necroptosis, microglial polarization, and cognitive function. Second, we did not validate the protective effects of TAK-242 on microglial polarization *in vivo*. Future studies should focus on *in vivo* models to confirm the role of TLR4/MyD88 in neuroinflammation and cognitive impairment and further explore its therapeutic potential. In the Morris water maze, we will measure parameters such as the latency to find the hidden platform, swimming paths, and the time spent in the target quadrant to evaluate spatial learning and memory abilities of mice in different treatment groups. In the novel object recognition test, we will record the exploration time differences between novel and familiar objects to assess the cognitive recognition capabilities of mice. These behavioral experiments will provide direct and quantitative data on PTX-induced cognitive deficits and the improvements achieved by interventions like Nec-1 treatment, thereby strengthening the clinical significance of our findings. Finally, we solely relied on pharmacological inhibitors in our experiments. While these inhibitors are well established in the field, potential off-target effects cannot be completely ruled out. In future research, we plan to employ siRNA or other genetic approaches, such as CRISPR/Cas9 gene editing, to target genes like RIPK1, TLR4, and MyD88.

## 5 Conclusion

In summary, this study demonstrated that hippocampal neuron necroptosis activates microglia through the TLR4/MyD88 signaling pathway in PTX-induced cognitive impairment, promoting M1 polarization and neuroinflammation, while inhibiting necroptosis promotes M2 polarization and neuroprotection. We also observed an increase in M2 microglia and BDNF expression after inhibiting hippocampal neuron necroptosis. However, whether this increase in M2 microglia, through secreting higher levels of BDNF, can directly promote neuronal survival and repair and lead to cognitive function improvement remains a hypothesis that requires further verification in future studies.
